# TP0586532, a Novel Non-Hydroxamate LpxC Inhibitor: Potentiating Effect on *In Vitro* Activity of Meropenem against Carbapenem-Resistant Enterobacteriaceae

**DOI:** 10.1128/spectrum.00828-22

**Published:** 2022-06-01

**Authors:** Ippei Yoshida, Iichiro Takata, Kiyoko Fujita, Hajime Takashima, Hiroyuki Sugiyama

**Affiliations:** a Pharmacology Laboratories, Taisho Pharmaceutical Co., Ltd., Saitama, Japan; b Chemistry Laboratories, Taisho Pharmaceutical Co., Ltd., Saitama, Japan; c Medical Information, Taisho Pharmaceutical Co., Ltd., Tokyo, Japan; Louis Stokes Cleveland VAMC

**Keywords:** carbapenem-resistant Enterobacteriaceae, LpxC inhibitor, TP0586532, combination, meropenem, permeability, potentiate

## Abstract

Carbapenem-resistant Enterobacteriaceae (CRE) are an urgent threat to public health requiring the development of novel therapies. TP0586532 is a novel non-hydroxamate LpxC inhibitor that inhibits the synthesis of lipopolysaccharides, which are components of the outer membranes of Gram-negative bacteria. Based on the mechanism of action of TP0586532, we hypothesized that it might enhance the antibacterial activity of other antibiotics by increasing the permeability of the outer bacterial membrane. The combination of TP0586532 with meropenem, amikacin, cefepime, piperacillin, and tigecycline showed synergistic and additive effects against carbapenem-susceptible Klebsiella pneumoniae and Escherichia coli. Checkerboard experiments against 21 carbapenem-resistant K. pneumoniae and E. coli strains (13 *bla*_KPC_+, 5 *bla*_NDM-1_+, 2 *bla*_VIM_+, and 1 *bla*_IMP_+) showed that the combination of TP0586532 with meropenem yielded synergistic and additive effects against 9 and 12 strains, respectively. In a time-kill assay examining 12 CRE strains, synergistic effects were observed when TP0586532 was combined with meropenem against many of the strains. A membrane permeability assay using ethidium bromide (EtBr) was performed to investigate the mechanism of the potentiating effect. TP0586532 increased the influx of EtBr into a CRE strain, suggesting that TP0586532 increased membrane permeability and facilitated intracellular access for the antibiotics. Our study demonstrates that TP0586532 potentiates the *in vitro* antibacterial activity of meropenem against CRE. Combination therapy consisting of TP0586532 and meropenem has potential as a treatment for CRE infections.

**IMPORTANCE** Carbapenem-resistant Enterobacteriaceae (CRE) are an urgent public health threat, as therapeutic options are limited. TP0586532 is a novel LpxC inhibitor that inhibits the synthesis of lipopolysaccharides in the outer membranes of Gram-negative bacteria. Here, we demonstrated the potentiating effects of TP0586532 on the antibacterial activity of meropenem against CRE harboring various types of carbapenemase genes (*bla*_KPC_+, *bla*_NDM-1_+ *bla*_VIM_+, and *bla*_IMP_+). TP0586532 also augmented the bactericidal effects of meropenem against CRE strains, even against those with a high level of resistance to meropenem. The potentiating effects were suggested to be mediated by an increase in bacterial membrane permeability. Our study revealed that a combination therapy consisting of TP0586532 and meropenem has the potential to be a novel therapeutic option for CRE infections.

## INTRODUCTION

The prevalence of carbapenem-resistant Enterobacteriaceae (CRE) has increased worldwide, with the Centers for Disease Control and Prevention (CDC) classifies it as an urgent threat, the highest level of concern to human health (1). CRE infections requiring hospitalization are estimated to be as high as 2.7 to 3.1 million worldwide (2), and the mortality rates for CRE infections are higher than those for carbapenem-susceptible Enterobacteriaceae (CSE) infections (3, 4). The reasons for the severity of CRE infections are limited therapeutic options and delays in appropriate therapy (3, 4). Until several years ago, antibiotics that could be used to treat CRE infections were extremely limited, with polymyxins (colistin and polymyxin B) being available in some cases (5). However, polymyxins are associated with a high risk of nephrotoxicity. Furthermore, the colistin-resistance rate of CRE is reportedly higher than that of CSE, presumably because of the increasing use of colistin to treat CRE infections (6). In recent years, new antibiotics such as ceftazidime-avibactam, meropenem-vaborbactam, and plazomicin have been approved, and these antibiotics have been shown to be more effective against CRE infections and less nephrotoxic than polymyxins (7–9). However, CRE clinical isolates resistant to these antibiotics have already been reported (10–12). Accordingly, additional therapeutic strategies for CRE infections are urgently needed.

In medical guidelines, combination therapy is empirically recommended for the treatment of severe infections, such as septic shock, ventilator-associated pneumonia, and high-risk hospital-acquired pneumonia (13, 14). Recently, reports of the *in vitro* efficacy of antimicrobial combinations against CRE have been increasing. Notably, the use of carbapenems in combination with other antibiotics has shown bactericidal effects against CRE strains, even when the individual antibiotics do not have bactericidal effects when used alone (15–18). In addition, various clinical data have indicated that combination therapies are useful for the treatment of CRE infections, especially bacteremia (19–23). Cohort studies of patients with bloodstream infections caused by carbapenemase-producing Klebsiella pneumoniae have shown that the mortality rate after combination therapy, especially with carbapenem-containing combinations, is significantly lower than that after monotherapy (19, 20). These findings suggest that a combination therapy including carbapenem is useful for the treatment of CRE infections.

**FIG 1:**
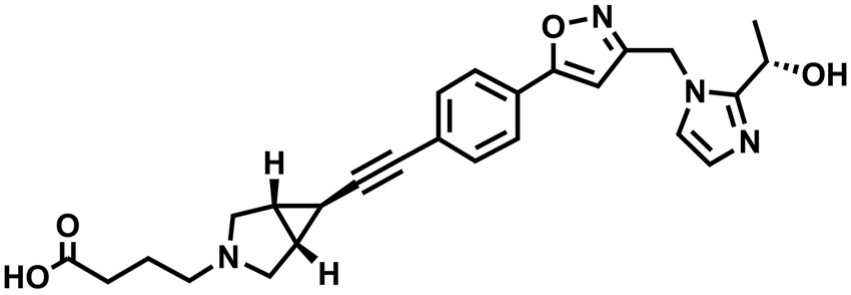
Chemical structure of TP0586532.

UDP-3-*O*-acyl-*N*-acetylglucosamine deacetylase (LpxC) is an enzyme that catalyzes the synthesis of lipopolysaccharide (LPS), an outer membrane component in Gram-negative bacteria (24). Although ACHN-975, a hydroxamate LpxC inhibitor, was evaluated in a clinical trial, this compound had cardiovascular toxicity (25). Hydroxamate is a robust zinc ion chelator and could lead to unwanted side effects by inhibiting several human metalloenzymes (26). Therefore, we have made efforts to identify a non-hydroxamate LpxC inhibitor. TP0586532 (Fig. 1) is a novel non-hydroxamate LpxC inhibitor that shows antibacterial activity against Gram-negative bacteria, including CRE (27). Furthermore, TP0586532 had no effect on blood pressure, heart rate, or electrocardiogram findings in a cardiovascular study examining anesthetized guinea pigs (28). In this study, to investigate the potential of TP0586532 to enhance the antibacterial activities of other antibiotics, we assessed the antibacterial activities of various antibiotics used alone or with TP0586532 against Enterobacteriaceae using a checkerboard assay. In addition, time-kill experiments to examine the potentiating effects of TP0586532 on the *in vitro* bactericidal activity of meropenem against CRE were performed. Finally, the mechanism of this effect was examined.

## RESULTS

### Antibiotic-enhancing activity of TP0586532 on antibacterial activity.

Checkerboard assays were performed using all the tested antimicrobials in combination with TP0586532 against two Enterobacteriaceae strains. Combining TP0586532 with the antibiotics caused 2- to 8-fold decreases in their minimum inhibitory concentrations (MICs) against K. pneumoniae ATCC 13883. The fractional inhibitory concentrations indices (FICIs) of TP0586532 when used with the antibiotics against K. pneumoniae ATCC 13883 ranged from 0.375 to 0.625; in other words, all the combinations provided a synergistic or additive effect (Table 1). Against Escherichia coli ATCC 25922, the combination of TP0586532 with the antibiotics caused a 2- to 32-fold decrease in the MICs of the antibiotics. Synergistic or additive effects were observed for the combinations of TP0586532 with all of the tested antibiotics except for ciprofloxacin and colistin, with FICIs ranging from 0.370 to 0.750 (Table 2). The effect of TP0586532 used in combination with ciprofloxacin or colistin against E. coli ATCC 25922 were indifferent from that of TP0586532 alone. Antagonism between TP0586532 and any of the antibiotics was not seen.

**TABLE 1:** FICIs of antibacterial drugs used in combination with TP0586532 against K. pneumoniae ATCC 13883^*a*^

Antibiotic	MICs (μg/mL)				FICI	Outcome^*b*^
	Antibiotic		TP0586532			
	Alone	Combination	Alone	Combination		
MEM	0.12	0.03/0.015^*c*^	4	0.5/1^*c*^	0.375	Synergistic
AMK	1	0.25	4	1	0.500	Synergistic
FEP	0.12	0.06	4	0.5	0.625	Additive
CIP	0.06	0.03	4	0.12	0.530	Additive
CST	2	1	4	0.5	0.625	Additive
PIP	32	8	2	0.5	0.500	Synergistic
TGC	1	0.5	4	0.5	0.625	Additive

a MEM, meropenem; AMK, amikacin; FEP, cefepime; CIP, ciprofloxacin; CST, colistin; PIP, piperacillin, TGC, tigecycline; MIC, minimum inhibitory concentration; FICI, fractional inhibitory concentration index.

b FICI interpretation: ≤0.5, synergistic; >0.5 to ≤1, additive.

c Two MIC pairs of TP0586532 and MEM had the same FICI.

**TABLE 2:** FICIs of antibacterial drugs used in combination with TP0586532 against E. coli ATCC 25922^*a*^

Antibiotic	MICs (μg/mL)				FICI	Outcome^*b*^
	Antibiotic		TP0586532			
	Alone	Combination	Alone	Combination		
MEM	0.03	0.015	1	0.25	0.750	Additive
AMK	4	1	1	0.12	0.370	Synergistic
FEP	0.06	0.03	2	0.12	0.560	Additive
CIP	0.008	0.00025	1	1	1.03	Indifferent
CST	0.5	0.015	1	1	1.03	Indifferent
PIP	4	0.5	1	0.25	0.375	Synergistic
TGC	0.25	0.12	1	0.12	0.600	Additive

a MEM, meropenem; AMK, amikacin; FEP, cefepime; CIP, ciprofloxacin; CST, colistin; PIP, piperacillin, TGC, tigecycline; MIC, minimum inhibitory concentration; FICI, fractional inhibitory concentration index.

b FICI interpretation: ≤0.5, synergistic; >0.5 to ≤1, additive; >1 to ≤2, indifferent between antibiotic alone or antibiotic + TP0586532 combination.

Carbapenem-containing combinations are reportedly effective against CRE both *in vitro* (15–18) and in clinical settings (19, 20). We therefore investigated whether meropenem plus TP0586532 was effective against 21 CRE strains (13 *bla*_KPC_+, 5 *bla*_NDM-1_+, 2 *bla*_VIM_+, and 1 *bla*_IMP_+) using a checkerboard assay. The MICs of meropenem when used in combination with TP0586532 at concentrations of 0.125 to 0.5 × MIC decreased by 2- to 512-fold compared to the MICs of meropenem alone (Table 3). This combination exhibited synergistic or additive effects, but neither indifferent nor antagonistic effects, against all the CRE strains tested. Synergistic effects were observed against 9 K. pneumoniae strains, and additive effects were observed against 7 K. pneumoniae and 5 E. coli strains. These results revealed that TP0586532 potentiates the antibacterial activity of meropenem against CRE harboring various types of carbapenemase genes.

**TABLE 3:** FICIs of meropenem used in combination with TP0586532 against carbapenem-resistant Enterobacteriaceae^*a*^

Strain	Resistance gene	MICs (μg/mL)				FICI	Outcome^*b*^
		MEM		TP0586532			
		Alone	Combination	Alone	Combination		
K. pneumoniae							
ATCC BAA-1705	*bla*_KPC_	64	32	2	1	1.00	Additive
ATCC BAA-1898	*bla*_KPC-2_	64	32	2	0.5	0.750	Additive
ATCC BAA-1899	*bla*_KPC-2_	128	0.25	4	2	0.502	Additive
ATCC BAA-1900	*bla*_KPC-3_	32	4	4	1	0.375	Synergistic
ATCC BAA-1902	*bla*_KPC-3_	64	2	2	1	0.531	Additive
ATCC BAA-1903	*bla*_KPC-2_	32	8	4	1	0.500	Synergistic
ATCC BAA-1904	*bla*_KPC-3_	16	0.25	2	1	0.516	Additive
ATCC BAA-1905	*bla*_KPC-2_	32	8	2	0.5	0.500	Synergistic
ATCC BAA-2078	*bla*_KPC_	32	8	2	0.5	0.500	Synergistic
ATCC BAA-2342	*bla*_KPC_	32	8	2	0.5	0.500	Synergistic
ATCC BAA-2343	*bla*_KPC_	32	8	1	0.25	0.500	Synergistic
ATCC BAA-2344	*bla*_KPC_	64	32/16^*c*^	2	0.5/1^*c*^	0.750	Additive
ATCC BAA-2470	*bla*_NDM-1_	64	16	2	0.5	0.500	Synergistic
ATCC BAA-2578	*bla*_NDM-1_	64	16	2	0.5	0.500	Synergistic
NCTC 13439	*bla*_VIM-1_	32	1	4	2	0.531	Additive
NCTC 13440	*bla*_VIM-1_	64	16/8^*c*^	2	0.25/0.5^*c*^	0.375	Synergistic
							
E. coli							
ATCC BAA-2340	*bla*_KPC_	16	8	2	1	1.00	Additive
ATCC BAA-2452	*bla*_NDM-1_	64	0.5	1	0.5	0.508	Additive
ATCC BAA-2469	*bla*_NDM-1_	64	2	2	1	0.531	Additive
ATCC BAA-2471	*bla*_NDM-1_	128	64	1	0.5	1.00	Additive
NCTC 13476	*bla*_IMP_	16	8	1	0.5	1.00	Additive

a MEM, meropenem; MIC, minimum inhibitory concentration; FICI, fractional inhibitory concentration index.

b FICI interpretation: ≤0.5, synergistic; >0.5 to ≤1, additive.

c Two pairs of MICs against K. pneumoniae ATCC BAA-2344 and K. pneumoniae NCTC 13440 had the same FICI, respectively.

### Potentiating effect of TP0586532 on bactericidal activity of meropenem against CRE.

**FIG 2:**
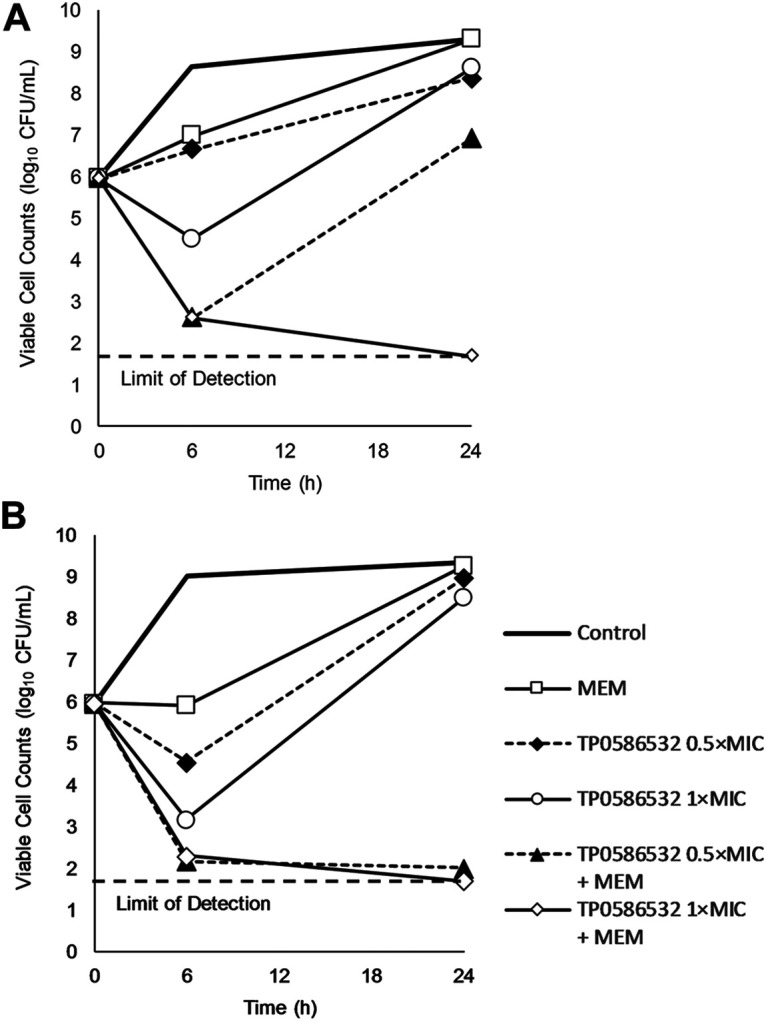
Time-kill curves of meropenem used in combination with TP0586532 against carbapenem-resistant K. pneumoniae ATCC BAA-1902 (A) and E. coli ATCC BAA-2469 (B). The bacteria were treated with meropenem (MEM, 8 μg/mL) alone or in combination with TP0586532 at 0.5 or 1 × minimum inhibitory concentration (MIC).

We investigated the potentiating effect of TP0586532 on the bactericidal activity of meropenem against CRE using a time-kill assay. Meropenem alone at 8 μg/mL did not reduce viable cell counts of K. pneumoniae ATCC BAA-1902 (Fig. 2A). In contrast, its combination with TP0586532 at 0.5 × MIC produced a synergistic and bactericidal effect at 6 h. In addition, its combination with TP0586532 at 1 × MIC reduced the viable cell count to below the detection limit at 24 h. In E. coli ATCC BAA-2469, a temporary bacteriostatic effect followed by regrowth was observed for meropenem alone (Fig. 2B). However, when combined with TP0586532 at 0.5 and 1 × MIC, a bactericidal effect, but not regrowth, was observed at 24 h. The potentiating effect of TP0586532 on the bactericidal activity of meropenem was also evaluated against other CRE strains (Table 4). In total, 12 CRE strains were tested, and the combination treatment produced synergistic (10/12) or additive (1/12) effects. Furthermore, meropenem alone had no bactericidal effect against almost all the CRE strains, whereas its combination with TP0586532 produced bactericidal effects against the majority of the strains (9/12).

**TABLE 4:** Change in viable cell counts of meropenem-resistant Enterobacteriaceae treated with meropenem in combination with TP0586532^*a*^

Strain	MEM MIC (μg/mL)	Time (h)	Change compared to initial inoculum (log_10_ CFU/mL)^*b*^				
			Alone			Combination	
			MEM	TP 0.5	TP 1	MEM + TP 0.5	MEM + TP 1
K. pneumoniae							
ATCC BAA-1900	32	6	−0.01	−0.36	−1.61	−2.46	−2.38
		24	3.61	3.46	3.50	−0.07	−0.27
ATCC BAA-1902	64	6	1.02	0.69	−1.45	**−3.35**	−**3.35**
		24	3.32	2.40	2.63	0.95	−**4.26**
ATCC BAA-1904	16	6	−1.81	0.71	−1.11	−**3.62**	−0.38
		24	3.53	3.21	3.31	−**3.74**	−0.49
ATCC BAA-2078	32	6	−0.73	0.63	−1.55	−**3.21**	−**3.21**
		24	3.32	3.32	3.28	1.71	−0.57
ATCC BAA-2343	32	6	−0.18	1.97	0.93	−0.86	−**3.72**
		24	3.02	2.79	2.79	2.50	2.66
ATCC BAA-2470	64	6	−1.58	1.66	0.38	−1.53	−0.91
		24	3.03	3.16	2.70	1.84	−1.70
ATCC BAA-2578	64	6	−1.39	0.90	0.21	−**3.50**	−**3.20**
		24	3.17	3.20	3.10	−0.86	−1.53
NCTC 13439	32	6	−1.33	−1.20	−1.37	−**3.68**	−1.92
		24	3.24	3.12	3.14	**<−4.28**	−1.02
NCTC 13440	64	6	−2.38	1.73	−0.16	−1.64	**−4.28**
		24	3.12	2.98	2.72	1.23	−**4.28**
							
E. coli							
ATCC BAA-2340	16	6	−1.96	0.00	−2.30	−2.10	−1.75
		24	−**3.27**	2.26	2.39	−**3.75**	−2.22
ATCC BAA-2452	64	6	−0.32	0.51	−1.18	−1.73	−2.99
		24	3.49	3.09	2.72	3.13	2.83
ATCC BAA-2469	64	6	−0.07	−1.43	−2.79	**−3.79**	**−3.67**
		24	3.29	2.98	2.51	−**3.97**	**−4.27**

a MEM, meropenem (8 μg/mL); MIC, minimum inhibitory concentration; TP 0.5, TP0586532 at 0.5 × MIC; TP 1, TP0586532 at 1 × MIC.

b The dark gray and light gray boxes indicate synergistic (≥2 log_10_ reduction) and additive (1 to <2 log_10_ reduction) effects when used in combination, compared with the effect of the most active single agent, respectively. Bold type indicates a bactericidal effect (≥3 log_10_ reduction compared with the initial inoculum).

### Increase in membrane permeability by TP0586532.

**FIG 3:**
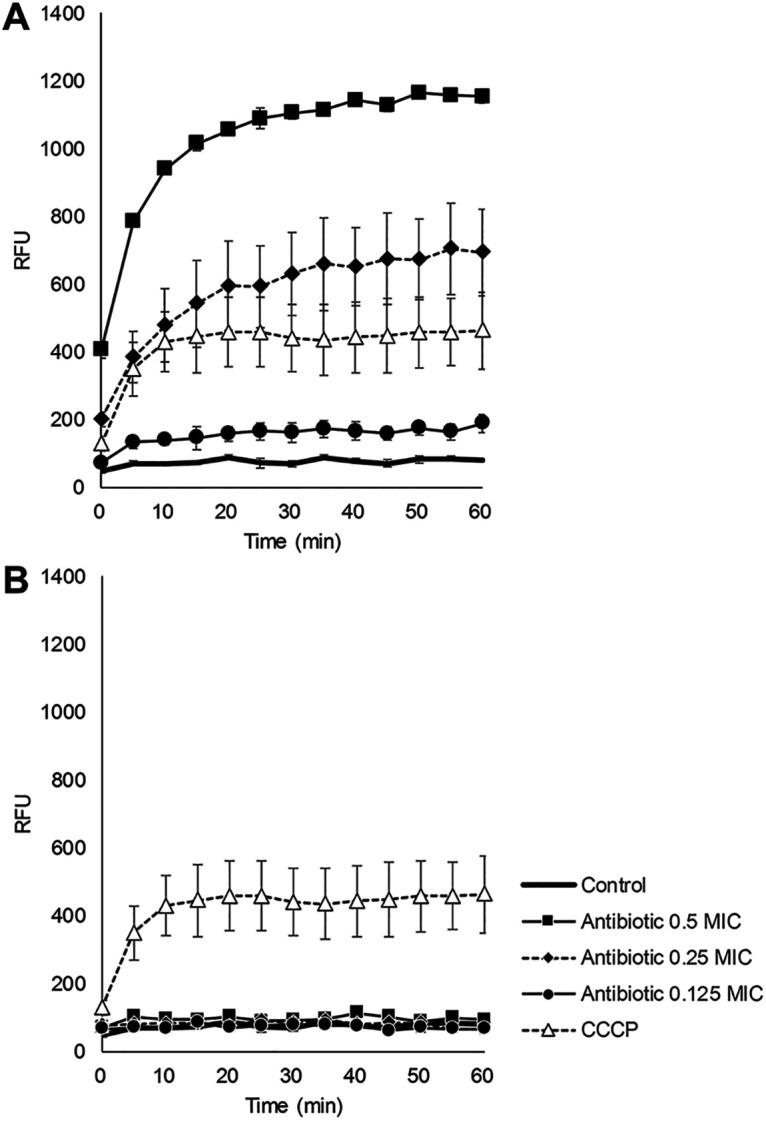
Membrane permeability of K. pneumoniae NCTC 13440 treated with TP0586532 (A) or azithromycin (B). The bacteria were treated with TP0586532 or azithromycin at 0.125, 0.25, or 0.5 × MIC for 2 h, and then prepared at 1 to 2 × 10^8^ CFU/mL in phosphate-buffered saline (PBS). The sample of carbonyl cyanide m-chlorophenyl hydrazone (CCCP) was prepared in PBS containing 20 μg/mL of CCCP without antibiotic treatment. Data are represented as the means ± standard error of the mean (SEM) of three independent experiments.

Since TP0586532 acts by inhibiting LpxC, an enzyme required for the synthesis of LPS, we assumed that TP0586532 increased the antibacterial activities of other antibiotics by increasing the permeability of the outer membrane. To investigate this hypothesis, an ethidium bromide uptake assay was performed on K. pneumoniae NCTC 13440. When bacteria were treated with carbonyl cyanide m-chlorophenyl hydrazone (CCCP), an ionophore which disperses the transmembrane proton gradient and has been previously used as a positive control for this assay (29), EtBr accumulated in the cytoplasm of bacteria, producing fluorescence (Fig. 3A and B). As expected, TP0586532 also increased the fluorescent intensity in a dose-dependent manner (Fig. 3A). Meanwhile, azithromycin, a protein synthesis inhibitor that has no effect on membrane permeability (30), did not increase the fluorescent intensity, similar to the no-treatment control (Fig. 3B). These results suggest that TP0586532 increases the membrane permeability of Gram-negative bacteria.

## DISCUSSION

The therapeutic effects of meropenem-containing regimens against CRE infections have been studied clinically (19–23). Among these studies, Daikos et al. (19) and Tumbarello et al. (20) revealed that carbapenem-containing treatments are useful against carbapenemase-producing K. pneumoniae infections, but only if the carbapenem MICs were ≤8 μg/mL. Our results showed that when TP0586532 was used in combination with antibiotics, it produced synergistic or additive effects against all CRE strains with a meropenem MIC of >8 μg/mL in the checkerboard assay. Furthermore, in a time-kill assay, meropenem at a clinically achievable concentration of 8 μg/mL (31) used in combination with TP0586532 exhibited synergistic or additive effects against a large number of CRE strains with a meropenem MIC of >8 μg/mL. A bactericidal effect was observed for the combination of meropenem with TP0586532 at 1 × MIC even against strains with an MIC of 64 μg/mL (K. pneumoniae ATCC BAA-1902, K. pneumoniae NCTC 13440, and E. coli ATCC BAA-2469), and this effect lasted for 24 h. Although regrowth was observed in some strains after the combination of meropenem with TP0586532, the bacterial burden of many of the strains at 24 h was lower than that after meropenem alone. Since TP0586532 potentiated the bactericidal activity of meropenem against CRE in a concentration-dependent manner, the combination treatment with a higher concentration of TP0586532 can more rapidly reduce bacterial load and, as a result, inhibit the development of resistance and regrowth. The maximum unbound plasma concentration (*fC*_max_) of TP0586532 in humans after administration of the clinically effective dose was estimated to be 13.1 μg/mL (32). This estimated concentration is 3.28-fold higher than the concentration at which regrowth was seen at 24 h. Thus, we expect that TP0586532 will reduce the frequency of resistance and regrowth in clinical settings.

Some synergy studies have shown no correlation with clinical outcomes; however, one of the reasons for this is that they were often performed using high concentrations not achievable in humans (33). On the other hand, the results of checkerboard assays using concentrations that can be achieved in humans have been reported to be correlated with those of time-kill assays and clinical outcomes (34). In our study, bactericidal effects against CRE strains were observed for combinations of TP0586532 and meropenem at each concentration achievable in humans, so combination therapy has the potential to exert bactericidal effects in clinical practice.

Considering the potentiating effects of TP0586532 on the *in vitro* activity of meropenem against CRE strains, TP0586532 has the potential to produce a clinically therapeutic effect when used in combination with meropenem against CRE infections, even in strains with a meropenem MIC of >8 μg/mL. Moreover, we have previously demonstrated that TP0586532 attenuated LPS release in K. pneumoniae and IL-6 production in a K. pneumoniae-infected lung induced by meropenem (35). Overall, from our studies, TP0586532 can not only potentiate the antibacterial activity of meropenem but also attenuate the inflammation induced by meropenem. However, this study only examined the potentiating effects of TP0586532 against CRE strains which harbor carbapenemase genes *in vitro*. Therefore, a combination study on non-carbapenemase-producing CRE to comprehensively understand the potentiating effects of TP0586532 is needed. Besides this, an animal model for estimation of human exposure is required to confirm clinical efficacy.

TP0586532 induced EtBr accumulation in carbapenem-resistant K. pneumoniae in the EtBr uptake assay. EtBr accumulation in bacterial cells has been demonstrated to be caused by increased membrane permeability (36) or the inhibition of efflux pumps (37). In addition, the loss of LPS expression reportedly causes increased membrane permeability in Acinetobacter baumannii, thereby increasing its susceptibility to antibiotics (36). Therefore, TP0586532 is considered to increase membrane permeability in carbapenem-resistant K. pneumoniae by inhibiting LPS synthesis and facilitating intracellular access by EtBr. In this manner, TP0586532 may increase the membrane permeability of the CRE strains and effectively potentiate the antibacterial activity of meropenem. The potentiation effects of TP0586532 were different among strains in the combination studies. This is speculated to be attributed to differences in levels of membrane permeability increase or other factors such as efflux pump activity and/or porin impermeability observed in CRE (38). Further detailed studies of each strain are expected to be useful to elucidate the mechanisms of the potentiation effects of TP0586532.

TP0586532 and colistin combinations showed indifferent or additive effects, and their effect was the weakest among all the tested antibiotics. TP0586532 might have a minimal effect on the antibacterial activity of colistin because colistin produces antibacterial activity by binding to LPS (39), whereas TP0586532 reduces LPS levels (27).

In conclusion, TP0586532 enhanced the antibacterial activity of various antibiotics, especially meropenem, against Enterobacteriaceae, including CRE. TP0586532 also augmented the bactericidal effects of meropenem against CRE. The potentiating mechanism was suggested to increase bacterial membrane permeability and facilitate meropenem intracellular access. This study demonstrates that meropenem used in combination with TP0586532 has therapeutic potential for the treatment of severe CRE infections, including strains with a high level of resistance to meropenem.

## MATERIALS AND METHODS

### Bacterial strains, culture media, and antibiotics.

K. pneumoniae and E. coli strains were purchased from the American Type Culture Collection (Manassas, VA) or the National Collection of Type Cultures (London, United Kingdom). Cation-adjusted Mueller-Hinton broth was used for antibacterial susceptibility testing and time-kill assays, and heart infusion agar was used for the enumeration of viable cells.

TP0586532 was synthesized by the Department of Medicinal Chemistry, Taisho Pharmaceutical Co., Ltd. (Saitama, Japan). Amikacin disulfate salt, colistin sulfate, piperacillin, tigecycline hydrate, and azithromycin dihydrate were purchased from Sigma-Aldrich (St. Louis, MO). Cefepime hydrochloride was purchased from US Pharmacopeia (Rockville, MD). Meropenem trihydrate were purchased from US Pharmacopeia or FUJIFILM Wako Pure Chemical Corporation (Osaka, Japan). Ciprofloxacin hydrochloride monohydrate was purchased from FUJIFILM Wako Pure Chemical Corporation.

### Checkerboard assay.

The MICs of antibiotics alone or in combination with TP0586532 were measured using the broth microdilution method according to Clinical and Laboratory Standards Institute (CLSI) guidelines (40). The FICI was determined using the following equation: FICI = MIC of compound A in combination with compound B/MIC of compound A alone + MIC of compound B in combination with compound A/MIC of compound B alone. The interaction was defined as synergistic if the FICI was ≤0.5, additive if FICI was >0.5 to ≤1, indifferent if FICI was >1 to ≤2, and antagonistic if FICI was >2 (41).

### Time-kill assay.

The bacterial strains were cultured at 35°C for 2 h, and the preculture was inoculated into fresh medium containing meropenem (8 μg/mL) alone or in combination with TP0586532 at 0.5 or 1 × MIC. The numbers of viable cells were determined by plating serial dilutions at 0, 6, and 24 h after inoculation. The limit of detection was 1.70 log_10_ CFU/mL. Synergy and additivity were defined as ≥2 log_10_ and 1 to <2 log_10_ reductions in the viable cell count when used in combination compared with that for the most active single agent, respectively (42). A bactericidal effect was defined as a ≥3 log_10_ reduction compared with the initial inoculum (42).

### EtBr uptake assay.

An EtBr uptake assay was performed using a protocol modified from a previous report (36). The bacterial strains were cultured at 35°C for 2 h, and the preculture was inoculated into fresh medium containing 0.125, 0.25, or 0.5 × MIC of TP0586532 or azithromycin. After 2 h of incubation at 35°C, the bacteria were centrifuged at 10,000 × *g* for 1 min. The pellet was washed with phosphate-buffered saline: typo (PBS) and adjusted with 1 to 2 × 10^8^ CFU/mL in PBS or PBS containing 20 μg/mL of CCCP. Next, EtBr was added to obtain a final concentration of 1 μg/mL, and fluorescence (λ_excite_ : 530 nm/λ_emit_ : 600 nm) was measured for 60 min.
